# Hitting the Target but Missing the Point? Modelling Health and Economic Impacts of Different Approaches to Meeting the Global Action Plan for Physical Activity Target

**DOI:** 10.1007/s40279-020-01398-2

**Published:** 2021-01-12

**Authors:** Anja Mizdrak, Ding Ding, Christine Cleghorn, Tony Blakely, Justin Richards

**Affiliations:** 1 Department of Public Health, University of Otago (Wellington), 23 Mein Street, Newtown, Wellington, New Zealand; 2 Prevention Research Collaboration, Faculty of Medicine and Health, Sydney School of Public Health, The University of Sydney, Camperdown, NSW Australia; 3 Charles Perkins Centre, The University of Sydney, Camperdown, NSW Australia; 4 Population Interventions, Melbourne School of Population and Global Health, University of Melbourne, Melbourne, Australia; 5 Faculty of Health, Victoria University Wellington, Wellington, New Zealand; 6 Sport New Zealand, Wellington, New Zealand

## Abstract

**Background:**

The World Health Organization launched the Global Action Plan for Physical Activity (GAPPA) in 2018, which set a global target of a 15% relative reduction in the prevalence of physical inactivity by 2030. This target, however, could be acheived in various ways.

**Methods:**

We use an established multi-state life table model to estimate the health and economic gains that would accrue over the lifetime of the 2011 New Zealand population if the GAPPA target was met under two different approaches: (1) an equal shift approach where physical activity increases by the same absolute amount for everyone; (2) a proportional shift approach where physical activity increases proportionally to current activity levels.

**Findings:**

An equal shift approach to meeting the GAPPA target would result in 197,000 health-adjusted life-years (HALYs) gained (95% uncertainty interval (UI) 152,000–246,000) and healthcare system cost savings of US$1.57b (95%UI $1.16b–$2.03b; 0% discount rate). A proportional shift to the GAPPA target would result in 158,000 HALYs (95%UI 127,000–194,000) and US$1.29billion (95%UI $0.99b–$1.64b) savings to the healthcare system.

**Interpretation:**

Achieving the GAPPA target would result in large health gains and savings to the healthcare system. However, not all population approaches to increasing physical activity are equal—some population shifts bring greater health benefits. Our results demonstrate the need to consider the entire population physical activity distribution in addition to evaluating progress towards a target.

**Supplementary Information:**

The online version contains supplementary material available at 10.1007/s40279-020-01398-2.

## Plain Language Summary

The World Health Organization launched the Global Action Plan for Physical Activity in 2018, which set a global target to reduce physical inactivity. We explored different ways in which this target could be met and found that some approaches to meeting the target would bring larger health gains and savings to the healthcare system than others.

**Supplementary Information:**

The online version contains supplementary material available at 10.1007/s40279-020-01398-2.

## Key Points

**Table:** 

Different population-level approaches to increasing physical activity have differing impacts on health gains and healthcare system costs.
Approaches that are equally effective at increasing activity across the entire population are likely to bring greater health improvements than approaches that are more effective among the already active.
Multiple metrics should be used for population surveillance and intervention evaluation to better monitor progress, particularly in groups that are the least active.

## Introduction

Physical activity has a wide range of positive impacts on health and wellbeing, including reduced risk of cardiovascular diseases, selected cancers, type-2 diabetes, cognitive decline, several mental health disorders and all-cause mortality [1–4]. The World Health Organization (WHO) recommends that adults accumulate at least 150 min of moderate activity or 75 min vigorous activity per week (or equivalent combinations of the two) [5]. Despite the known benefits, many people do not meet these recommendations.

Globally, over a quarter of the population are insufficiently active, with a higher and increasing prevalence of physical inactivity in high-income countries [6]. High prevalence of physical inactivity places a burden on population health, the healthcare system, and the economy [7, 8]. To address the high burden of physical inactivity, the WHO launched the Global Action Plan for Physical Activity (GAPPA) in 2018 and set a global target of a 15% relative reduction in the prevalence of physical inactivity by 2030 [9].

Prevalence-based measures are commonly used and are considered easy-to-interpret ways to quantify physical activity at the population level. However, prevalence-based measures tend to force individuals into the dichotomy of “sufficiently active” and otherwise, irrespective of the population distribution on a broader spectrum of zero to high levels of physical activity. A prevalence-based target, such as the GAPPA target, does not specify how to shift the population distribution of physical activity and so countries may adopt different approaches to meeting the target.

Different approaches to tackling a risk factor, such as physical activity, have different implications for population health. Population approaches have long been recommended over targeted individual approaches to promote population health [10]. However, the extent to which the health impact of different population-level approaches differ is less clear. Comparing the population health and economic impact of different approaches to tackling physical inactivity is important for priority setting and resource allocation. Based on current epidemiological evidence, the greatest risk reduction comes from shifting individuals doing no activity to doing some, even if they are still below the threshold of physical activity recommendations [11].

This study aims to compare the health and economic impact of meeting the GAPPA target under different scenarios using Aotearoa New Zealand (NZ) as a case study. We contrast two approaches: an “equal shift” approach whereby everyone increases their physical activity by the same absolute amount, and a “proportional shift” approach whereby individuals’ increases in physical activity are proportional to their current activity levels. We estimated the annual physical activity increases required for NZ adults to meet the GAPPA target in 2030, and quantify the gains in population health (in health-adjusted life years (HALYs)) and healthcare system cost from the two approaches to meeting the target compared with a business-as-usual scenario.

## Methods

We modelled the health impact (in HALYs) and change in healthcare costs associated with meeting the GAPPA target. First, we estimated the increase in moderate-to-vigorous physical activity (MVPA) that would be required to shift the NZ adult population to meet the GAPPA target. Next, we used an established multi-state life-table model [12] to quantify the impact of incremental increases in physical activity required to meet the GAPPA target. Here, we outline relevant details about the Physical Activity and Active Transport Model (PAATM) used in this study and then move on to outline how we conceptualised the GAPPA target within the model structure. Further details are provided in Supplementary Materials S1, and additional information about the model has been published elsewhere [12, 13].

### Physical Activity and Active Transport Model

The Physical Activity and Active Transport Model (PAATM) is a proportional multi-state life table model that simulates population cohorts over time under different scenarios [12]. In this study, modelled scenarios represent changes in physical activity over time. These changes in physical activity are combined with relative risks to result in changes in disease incidence, which then lead to changes in the overall mortality and morbidity experience of the cohort. Modelled cohorts representing the entire NZ population in 2011 are defined by 5-year age groups, gender, and ethnicity (Māori—the indigenous population of NZ, and non-Māori). PAATM simulates these cohorts over the remainder of their life course (or until age 110), in annual time steps.

PAATM is parameterised to New Zealand with high-quality national-level epidemiological and population data from relevant administrative sources. This includes disease rates from mortality and other health records (including hospitalisations and pharmaceutical records), morbidity (i.e. disability rates associated with disease states), and healthcare system costs associated with incidence, prevalence, and last 6 months of life for each disease, and costs for those who are healthy (i.e. absent of modelled disease states). Changes in healthcare system costs within the model result from changes in the proportions of the population in different disease states over time. Healthcare system costs are derived directly from health service usage data capturing publicly funded events, including hospitalisation, outpatient, pharmaceutical, laboratory testing, and primary care [14, 15].

For this study, estimates of the health gains are presented in HALYs for the lifetime of the modelled population. Modelled healthcare system cost impacts were converted to 2019 US$ for the international readership. In the main analysis, no discounting was applied to future health gain nor to healthcare cost estimates to ensure comparability when presenting estimates at different time points in the future and to enable comparison with other scenarios modelled without discounting. Results showing the original 2011 NZ$ values and 3% discounted results are presented in Supplementary Materials S2 and S3.

PAATM captures the impact of changes in MVPA, consistent with relative risks used to generate potential impact fraction. Business-as-usual physical activity levels were dervied from the 2011 NZ Health Survey (NZHS), a representative national survey of adults aged 15 + years (*n* > 12,000) [16]. The NZHS uses the NZ Physical Activity Questionnaire, a validated adaptation of the International Physical Activity Questionnaire (IPAQ), to assess time spent engaging in MVPA [16, 17]. We fitted a lognormal distribution to the proportion of survey respondents in different physical activity categories to estimate physical activity prevalence by age, sex, and ethnicity. We assumed no change in the physical activity distribution over time in the business-as-usual, consistent with observed physical activity trends between the base year of the model (2011) and the most recent data.

### Conceptualisation of GAPPA Target

The GAPPA target specifies a 15% relative reduction in the prevalence of physical inactivity by 2030 [9]. We defined the cut-off for physical activity in line with global guidelines which state that adults should accumulate a minimum of 150 min of MVPA per week [5].

**Fig. 1:**
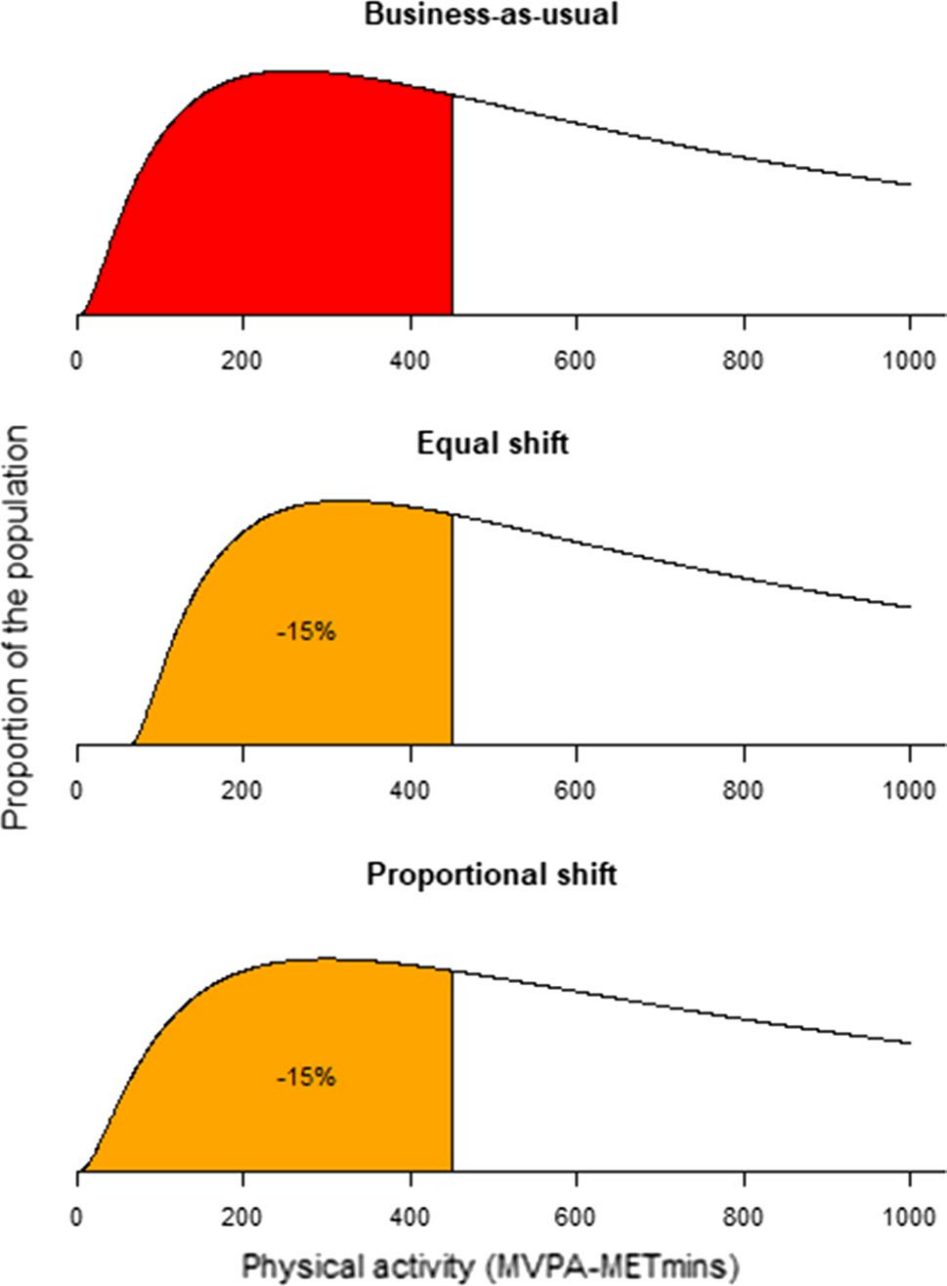
Conceptualisation of shift needed to meet GAPPA target, with shading representing the insufficiently active proportion of the population

Starting from the lognormal distribution of physical activity in the business-as-usual, the GAPPA target could be met through various shifts to the population distribution. We examined two scenarios that would achieve the GAPPA target by 2030 (illustrated in Fig. 1), selected to represent different population-level approaches: “equal shift”, and “proportional shift”.

In the “equal shift” approach, we assumed the GAPPA target would be met by an equal shift in physical activity that is applied evenly across the baseline physical activity distribution. This equates to all individuals in a cohort increasing their physical activity by the same amount so that the population meets the target. This scenario approximates a situation where a population intervention is equally effective for everyone in the population.

In the “proportional shift” approach, we assumed the GAPPA target would be met by shifting the mean of the lognormal distribution. In this scenario, increases in physical activity are proportionate to physical activity levels in the business-as-usual. For example, this scenario involves those with low levels of physical activity doing a little more and those engaging in high levels of physical activity doing a lot more. This scenario approximates a situation where a population intervention has an effect across the whole population, but the effect is greater (in absolute terms) for those who are already more active.

For both the scenarios, we first estimated the proportion (by age/sex/ethnicity) classified as insufficiently active at baseline, followed by estimating the proportion who would still be insufficiently active if the target was met as follows:

$$P_{{\text{target }}} = P_{{{\text{BAU}}}} - \left( {P_{{{\text{BAU}}}} \times 0.15} \right),$$

where *P* represents the proportion insufficiently active.

After calculating the total physical activity increase required to meet the target, we estimated the annual increase in physical activity that would be required to meet the target by 2030. We assumed a linear increase in physical activity from 2018 to 2030, with no change in physical activity between the base year of the model (2011) and 2018 under the GAPPA target scenario. In both scenarios, we assumed that physical activity prevalence post-2030 would remain constant (i.e. at the target level).

Each GAPPA target scenario was simulated 2000 times drawing probabilistically from the uncertainty distributions around each of the input parameters. The uncertainty intervals around the results represent the 2.5th and 97.5th percentiles of the 2000 simulations.

### Additional Analyses

We conducted additional analyses to estimate the health gain of under- and over-shooting the GAPPA target (10% and 20% reduction in the prevalence of insufficient physical activity respectively). For comparison with previous work examining the impact of eradicating tobacco and obesity [18], we also estimated the health gain of an ‘instant’ increase in physical activity to meet the target (i.e. full increase in physical activity is applied immediately), both for the ‘proportional shift’ and the ‘equal shift’ approaches. This scenario analysis was designed to assess the relative impact of increasing physical activity relative to other risk factors and inform the extent of additional health gains that would be possible with the immediate achievement of the GAPPA target.

## Results

### Characteristics of modelled population

At baseline (2011), 50.9% of the adult population were classified as insufficiently active. This would be reduced to 43.3% if the GAPPA Target of a 15% relative reduction in physical inactivity prevalence was achieved. Under the business-as-usual scenario, the modelled population accumulated a total of 172 million HALYs over the lifecourse.

### Health Gains

Over the lifetime of the modelled 2011 NZ population (*n* = 4,405,270), we estimate that 158,000 HALYs (95%UI 127,000–194,000) would be gained and the healthcare system would save US$1.29billion (95% UI $0.99b–$1.64b) from the proportional shift scenario. The health gains were higher under the equal shift approach, which resulted in 197,000HALYs gained (95%UI 152,000–246,000) and healthcare system cost savings of US$1.57b (95%UI $1.16b–$2.03b), also over the lifetime of the 2011 NZ population. This represents 18% higher health gains and 13% higher healthcare system savings in the “equal shift” approach compared to the “proportional shift” approach. The uncertainty intervals represent genuine uncertainty in modelled parameters that are common across the two modelled scenarios (e.g. disease rates). Regardless of the parameter values (randomly) chosen in each iteration of the model, the equal shift scenario results in higher health gains than the proportional shift scenario.

**Fig. 2:**
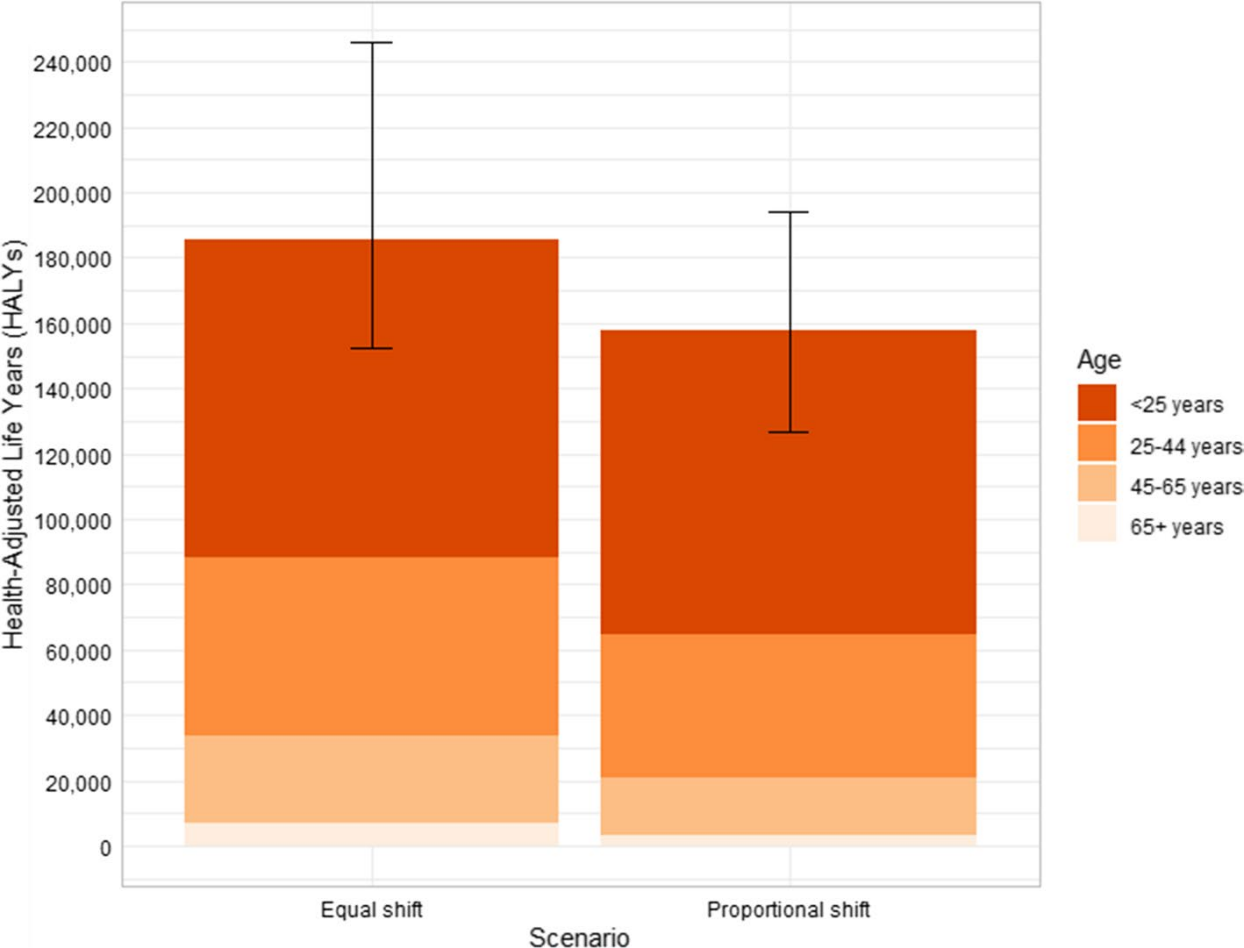
Health gains in health-adjusted life years from modelled scenarios (by age in 2011)

**Fig. 3:**
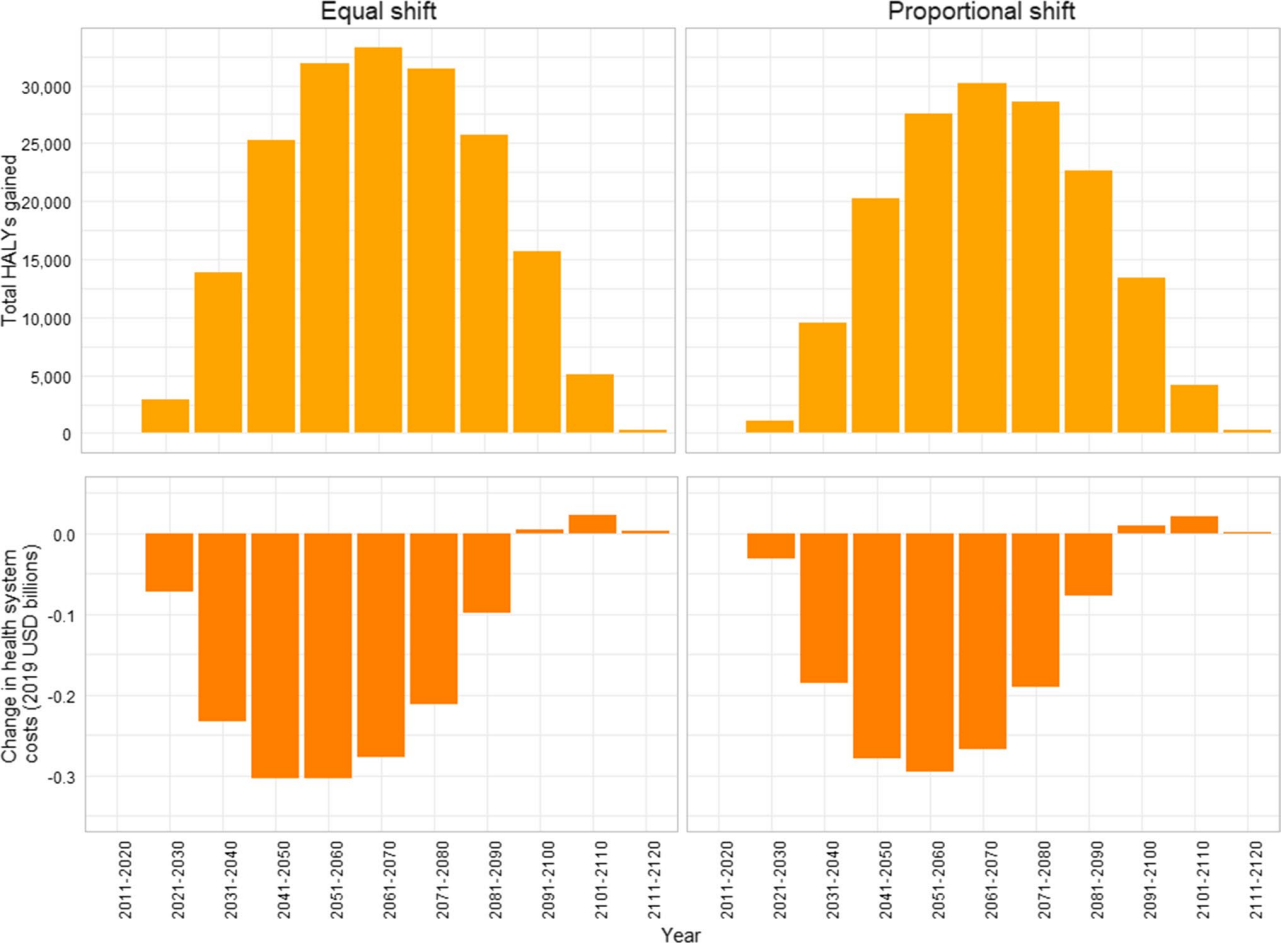
Health gains and change in the healthcare system costs over time under modelled scenarios

In both scenarios, nearly half of the total health gain is accumulated in the youngest age group (< 25 years in 2011) (Fig. 2). Health gains and healthcare system cost-savings peak mid-century (Fig. 3). This is due to the timing of the intervention resulting in younger age groups experiencing increases in physical activity for a longer period of time before reaching older ages when the modelled diseases tend to manifest.

### Additional Analyses

Predictably, the health gains would be reduced with a smaller (10%) relative reduction in physical inactivity prevalence (by a quarter for the equal shift scenario and a third for the proportional shift scenario), demonstrating the magnitude of health gains that would be achieved with partial progress towards the GAPPA target (see Table 1). Health gains would be higher than our main analysis if there were greater reductions in physical inactivity prevalence and more rapid implementation of the GAPPA target (see Table 1). Patterns for healthcare system costs were similar (see Supplementary Materials S4).

**Table 1:** Health-adjusted life-years gained over the lifetime of the 2011 NZ population under different scenario assumptions

	Equal shift	Proportional shift
Main result	185,000	158,000
10% relative reduction in physical inactivity prevalence	139,000	104,000
20% relative reduction in physical inactivity prevalence	224,000	213,000
Instant implementation of 15% GAPPA target	223,000	202,000

## Discussion

Achieving the GAPPA target would result in a substantial improvement in population health and cost saving to the healthcare system in NZ. The health impacts vary between the two scenarios examined, with the “equal shift” approach associated with greater health gains than the “proportional shift” approach. Our results highlight that not all population approaches are equal in terms of health gains and economic cost savings and that policy makers should consider implications of differences between approaches when setting targets, such as those outlined in the GAPPA.

Findings from our analysis complement earlier work examining the health and economic burdens of physical inactivity [7, 19]. While confirming the health and economic benefits of increasing population-level physical activity, our analysis expands the previous research through examining how to improve population-level physical activity and achieve the GAPPA target through different approaches. Whilst burden of disease studies provides valuable information on the consequences of political inaction, such information alone is insufficient for informing policy actions. By using a cohort-based model, we are able to explore scenarios that represent shifting population distributions over time, accounting for time lags between increasing physical activity and accumulating health gains, and allow for competing mortality and morbidity (i.e. accounting for future morbidity resulting from increased longevity). Our results show that achieving the GAPPA target through improving everyone’s physical activity levels by the same amount could lead to 18% greater health gain and 13% greater healthcare system cost savings (over the lifetime of the modelled population) than achieving the target through greater physical activity increases in those who are already doing more activity. Our analysis provides information on the likely magnitude and timing of health gains with a gradual increase to meet the GAPPA Target—an ambitious but realistic target. We encourage future work that examines the extent to which specific actions recommended in the GAPPA are likely to contribute to increasing physical activity and improving health in populations that participate in different volumes of activity at the outset.

At the national level, our study provides an estimate of how meeting the GAPPA Target compares to other public health interventions, policies and targets. We find that the physical activity increase required to meet the GAPPA Target in the NZ context is similar to the physical activity increases that would be observed by switching 25–50% of short trips (i.e. trips < 5 km) to walking and cycling [13]. In addition, we find that meeting the GAPPA Target would have a comparable impact to a > 90% reduction in the number of tobacco outlets [144,000QALYs (95% UI 75,800–247,000QALYs)], also modelled over the life course of the 2011 NZ population with 0% discounting [20].

### Strengths and limitations

The modelled scenarios represent two possible approaches to shifting the distribution of physical activity to meet the GAPPA Target. In reality, actual population shifts in physical activity are unlikely to be as smooth as the transitions modelled here. However, we believe that the modelled scenarios clearly demonstrate differences in the long-term health impact of different population-level approaches to physical activity promotion.

The use of an established multi-state life table approach means that we are able to include a temporal dimension in the modelled scenarios. We examine the likely timing of health gains and healthcare system cost savings, a considerable advance on previous research where models were not well suited to examining temporal components or reporting the timing of health and cost impacts [21].

Our analysis provides a detailed examination of increasing physical activity at a national level. The availability of high-quality health and health cost data in NZ is a considerable strength of this study. However, baseline physical activity was assessed using a survey that required participants to recall physical activity in ten minute bouts [16]. This resulted in poor exposure assessment for participants doing small amounts of physical activity (i.e. bouts under 10 min). Therefore, we were unable to model a scenario that involves shifting those who do zero physical activity (i.e., a “high risk” approach). Despite this limitation, the greater health gain in the “equal shift” scenario demonstrates the importance, in terms of health gains and healthcare cost savings, of increasing physical activity among those who are the least active. Although our analysis clearly demonstrates the value of including the least active people in efforts to increase population levels of physical activity, it does not consider the relative difficulty and cost of changing their behaviour. This would require a cost–benefit analysis, which was not the focus of this paper, but is an approach that may warrant further investigation [22].

### Policy implications

Different approaches to increasing physical activity are likely to result in different levels of health and economic gain. This study draws on the work of Geoffrey Rose who pioneered understanding about the potential for population-level approaches to prevent disease [10]. Rose argued that population approaches to prevention result in greater health gains than a targeted approach focused only on those at highest risk. We extend Rose’s ideas to examine differences in health gains associated with different population approaches towards increasing physical activity. We estimate health gains to be greater under an “equal shift” approach whereby individuals in a cohort all experience the same absolute increase in physical activity. Our findings are unsurprising given that dose–response relationships between physical activity and health outcomes show that those with the lowest baseline levels of physical activity have the most to gain from increased activity [1, 2]. To our knowledge, this is the first study that compares different population approaches to physical activity promotion and has important implications for priority setting and policy making.

Our results underline the importance of considering the entire physical activity distribution in research and policy. Global assessments have found a stable prevalence of “sufficient” physical activity over time [6]. However, a stable prevalence of “sufficient” activity does not preclude the possibility of decreased burdens of inactivity and vice versa. Hypothetically, if Country X adopted interventions to successfully shift those who were doing zero physical activity to doing a small (albeit still insufficient) amount, Country X would experience population health gains but still not hit the GAPPA target. Conversely, Country Y may hit the GAPPA target solely by shifting those who were doing 140–149 min/week of physical activity to doing 150 min, but experience less health gains than Country X, and thereby somewhat ‘miss the point’ of using physical activity as a way to promote health and wellbeing. Similarly, interventions that appear effective at increasing the proportion of people who are “sufficiently” active may unexpectedly exacerbate health inequities. Differential impacts of interventions according to baseline levels of physical activity have been observed in evaluations; some interventions reach those who are already more physically active and fit [23], whilst others appeal more [24] or would bestow greater benefit [25] for previously inactive individuals. From the small number of examples where differential impacts by baseline physical activity have been assessed, interventions that are non-competitive and embedded in settings where inactive people are already present appear to better reach those who are not currently active. Our results reinforce the value of a population-wide shift in physical activity and the need to invest in a complex suite of interventions at scale to improve the levels of physical activity of the entire population. From a policy perspective, this involves genuine investment in initiatives that are adapted to different local communities and genuine cross-government collaboration to ensure that relevant socio-cultural, interpersonal and intrapersonal barriers to participating in physical activity are addressed.

We also encourage the use of multiple metrics for population surveillance and intervention evaluation to better capture physical activity distribution. This could include regular reporting on the proportion of people participating no physical activity and the proportion reporting up to half the recommended amount of physical activity in addition to the proportion meeting physical activity recommendations. We also encourage intervention evaluations to consider differential impacts of interventions by baseline physical activity level to better understand whose physical activity is changing. This approach would address an often neglected part of the current global physical activity recommendations that is not accounted for in the GAPPA target: “Inactive people should start with small amounts of physical activity” [5]. It also aligns with wider calls to better reflect the complexity of physical activity in surveillance, including measurement of physical activity domain, intensity, and related behaviours (e.g. sedentary behaviours, sleep) [26, 27].

## Conclusion

We estimate that achieving the GAPPA target of a 15% relative reduction in the prevalence of physical inactivity would result in large health gains and savings to the healthcare system. We find that different approaches to meeting the GAPPA target vary in the health gains that they are likely to achieve—not all population approaches to increasing physical activity are equal. Changing the overall distribution of physical activity, and not merely progressing towards a target, needs to be considered to maximise the health and economic gains from increasing physical activity.

## Supplementary Information

Below is the link to the electronic supplementary material.

Supplementary file1 (DOCX 57 KB)

## Declarations

### Availability of data and material

Model input data is available in the Physical Activity and Active Transport Model Technical Report [12].

### Code availability

The model version used to undertake the analyses described in this paper is available on request from the authors.
