# Supplementary material for: Hitting the Target but Missing the Point? Modelling Health and Economic Impacts of Different Approaches to Meeting the Global Action Plan for Physical Activity Target
Source: Sports Med. 2021 Jan 12;51(4):815–23. doi: 10.1007/s40279-020-01398-2 (PMC7981302; doi:10.1007/s40279-020-01398-2)
Supplement: Supplementary file 1 — Supplementary file1 (DOCX 57 KB) [file 40279_2020_1398_MOESM1_ESM.docx]

# Supplementary Materials

Mizdrak A*, Ding D, Cleghorn C, Blakely T, Richards J. Hitting the target but missing the point? Modelling health and economic impacts of different approaches to meeting the Global Action Plan for Physical Activity Target. *Sports Medicine*.

*Corresponding author. Affiliation: Department of Public Health, University of Otago (Wellington). Email: [anja.mizdrak@otago.ac.nz](mailto:anja.mizdrak@otago.ac.nz).

## S1: Additional detail on the model and scenarios

The Physical Activity and Active Transport Model (PAATM) was originally designed to model a variety of physical activity and transport-related interventions and includes nine diseases. For this study, disease states not directly linked to physical activity (i.e. those linked only to air pollution, and road injuries) experienced no change in the GAPPA Target scenarios compared to business-as-usual. Diseases are modelled independently, with the exception of type 2 diabetes, which was treated as a disease as well as a risk factor for coronary heart disease and stroke in the model.^1-3^ A conceptual diagram of the model is provided in Figure 1.

## Figure 1: Conceptual diagram of model (adapted from Mizdrak et al^3^)

CHD

Stroke

Colorectal cancer

Breast cancer

Lung cancer†

COPD†

LRTI†

Road injuries†

Physical activity (MVPA-METmins/week)

Type 2 Diabetes

DISEASE LIFE TABLES

GAPPA Target

Health system

costs

Health adjusted life years (HALYs)

OUTPUTS

†No change in disease in this study

GAPPA: Global Action Plan for Physical Activity

MET: Metabolic Equivalent of Task

MVPA: Moderate-to-Vigorous Physical Activity

Main Life Table

The overall mortality and morbidity experience of the modelled cohort is represented using a life table – with separate life tables for business-as-usual and counterfactual scenarios. Alongside the main life table are independent disease life tables; these represent the proportion of the cohort in different disease states (healthy, diseased, dead) and are a function of incidence, remission (for diseases where this applies), and case fatality.

The mortality and morbidity of the cohort under counterfactual scenarios is estimated as follows. First, changes in population-level physical activity are combined with relative risks (representing the dose-response relationship between physical activity and selected disease outcomes^4,5^) to generate potential impact fractions.^6^ The potential impact fractions change disease incidence. As changes in disease incidence may occur a number of years after changes in a risk factor (in this case physical activity), PAATM includes time lags whereby the potential impact fractions averaged across previous years are used to modify disease incidence. Changes in disease incidence affect future disease-specific prevalence and mortality, which in turn, change the overall mortality and morbidity experience of the modelled cohort.

For both the scenarios , we first estimated the proportion (by age/sex/ethnicity) classified as insufficiently active at baseline, followed by estimating the proportion who would still be insufficiently active if the target was met as follows:

$$P_{target}= P_{BAU}-\left( P_{BAU}*0.15 \right)$$

where P represents the proportion insufficiently active.

For the ‘proportional shift’ scenario, we used the Excel Solver add-in to estimate the increase in the mean required to achieve the GAPPA target. We set up the Excel Solver such that it minimised the absolute difference between the proportion insufficiently active as calculated from the distribution, and the GAPPA target proportion (i.e. $P_{target}$).

To calculate the required physical activity increase for the ‘equal shift’ scenario, we calculated the current physical activity level at $P_{target}$. The physical activity level at $P_{target}$ was deducted from the guideline amount to get the increase in physical activity that would be required (at the population level) for the target to be met by each cohort (i.e. by age, sex, and ethnicity).

## S2: Healthcare system cost impacts of modelled approaches over the lifetime of the 2011 NZ population (in 2011 NZD)

|  | Healthcare system costs, billions (UI) |
| --- | --- |
| Equal shift | -2.17 (-2.81 to -1.60) |
| Proportional shift | -1.79 (-2.27 to -1.36) |

NZD: New Zealand Dollar

UI: Uncertainty Interval

## S3: Health-adjusted life years gained and healthcare system costs over the lifetime of the 2011 NZ population using a 3% discount rate

|  | HALYs | Healthcare system costs, NZD 2011 billions | Healthcare system costs, USD 2019 billions |
| --- | --- | --- | --- |
| Equal shift | 41,100 | -0.61 | -0.44 |
| Proportional shift | 33,400 | -0.51 | -0.37 |

HALY: Health-adjusted life year

NZD: New Zealand Dollar

USD: United States Dollar

## S4: Healthcare system cost impacts over the lifetime of the 2011 NZ population under different scenario assumptions

|  | 2019 USD billions | | 2011 NZD billions | |
| --- | --- | --- | --- | --- |
|  | Equal shift | Proportional shift | Equal shift | Proportional shift |
| Main result | -1.81 | -1.71 | -2.50 | -2.36 |
| 10% relative reduction in physical inactivity prevalence | -1.49 | -1.32 | -2.06 | -1.82 |
| 20% relative reduction in physical inactivity prevalence | -1.09 | -0.85 | -1.51 | -1.18 |
| Instant implementation of 15% GAPPA target | -1.79 | -1.75 | -2.47 | -2.42 |

NZD: New Zealand Dollar

USD: United States Dollar

## References

1. Peters SAE, Huxley RR, Woodward M. Diabetes as a risk factor for stroke in women compared with men: a systematic review and meta-analysis of 64 cohorts, including 775 385 individuals and 12 539 strokes. *The Lancet* 2014; **383**(9933): 1973-80.

2. Peters SAE, Huxley RR, Woodward M. Diabetes as risk factor for incident coronary heart disease in women compared with men: a systematic review and meta-analysis of 64 cohorts including 858,507 individuals and 28,203 coronary events. *Diabetologia* 2014; **57**: 1542–51.

3. Mizdrak A, Blakely T, Cleghorn CL, Cobiac L. Technical Report for BODE3 Active Transport and Physical Activity Model: Department of Public Health, University of Otago, Wellington, 2018.

4. Wahid A, Manek N, Nichols M, et al. Quantifying the Association Between Physical Activity and Cardiovascular Disease and Diabetes: A Systematic Review and Meta-Analysis. *J Am Heart Assoc* 2016; **5**(9).

5. Kyu HH, Bachman VF, Alexander LT, et al. Physical activity and risk of breast cancer, colon cancer, diabetes, ischemic heart disease, and ischemic stroke events: systematic review and dose-response meta-analysis for the Global Burden of Disease Study 2013. *BMJ* 2016; **354**: i3857.

6. Barendregt JJ, Veerman JL. Categorical versus continuous risk factors and the calculation of potential impact fractions. *J Epidemiol Community Health* 2010; **64**(3): 209-12.
